# Prenatal stress improves the ability of neonatal lambs to maintain body surface temperature during periods of cold exposure

**DOI:** 10.1007/s00484-026-03243-z

**Published:** 2026-06-11

**Authors:** Lea Labeur, Alison H. Small, Sabine Schmoelzl

**Affiliations:** 1 CSIRO Agriculture, FD McMaster Laboratories, New England Highway, Armidale, NSW Australia; 2 School of Environmental and Rural Science, University of New England, Armidale, NSW Australia; 3 School of Agriculture, College of Environmental and Life Sciences, Food Futures Institute, Murdoch University, Murdoch, WA 6150 Australia

**Keywords:** Thermoregulation, Cold challenge, Neonate, Prenatal stress, Thermogenesis, Infrared thermography

## Abstract

Exposure to cold and hypothermia is a significant threat to newborn lamb survival in extensive systems, and the ability to thermoregulate is essential for improved survivability. Lambs exhibit enhanced thermogenic abilities and increased brown fat deposition when ewes experience cold during late-pregnancy. This study evaluated the effects of various prenatal stressors on newborn lamb thermogenesis through three experiments. Experiment 1 examined the impact of mid-pregnancy shearing and wetting on lamb thermogenesis. Experiment 2 involved transporting and cold-exposing late-pregnancy ewes. Experiment 3 combined stressors like yarding, transport, wetting, and cold exposure in mid- or late-pregnancy ewes. Four-hour-old lambs were subjected to a 1-hour cold challenge during which infrared thermal images from the dorsal midline were taken every 10 min. Lambs from ewes shorn and wetted during mid-pregnancy tended to have higher body surface temperatures than those handled during mid-pregnancy (Experiment 1; *P =* 0.073) and they also maintained body surface temperature better during the cold challenge (Experiment 1; *P =* 0.025). Lambs from ewes exposed to late-pregnancy combined stressors showed higher radiated body surface temperatures than both mid-pregnancy stressed and control lambs (Experiment 3; *P <* 0.05) while late-pregnancy stress group lambs maintained radiated body surface temperature longer than both mid-pregnancy stressed and control groups lambs (Experiment 3; *P <* 0.01). The study concludes that cold exposure combined with husbandry stressors leads to differences in lamb thermogenesis, with mechanisms varying based on the timing of stressors.

## Introduction

At birth lambs experience a rapid transition from the warm and protective womb to an external environment, creating a thermoregulatory challenge. Exposure to wind, rain, and low temperatures increase heat loss, heightening the risk of hypothermia (Alexander et al. 1980; Lynch et al. 1980). Consequently, heat production is a critical factor for lamb survival (Dwyer and Morgan 2006). Hypothermia-related deaths account for approximately 5–10% of lamb mortalities in Australia but can be substantially higher under certain conditions (Hughes et al. 1964; Refshauge et al. 2016).

Heat production in lambs is achieved by either shivering and muscular activity or through non-shivering thermogenesis, the latter relying on brown fat in peri-renal and inguinal regions (Alexander and Williams 1968; Symonds 2013). Non-shivering thermogenesis provides approximately 50% of neonatal lamb heat (Alexander and Williams 1968; Graña-Baumgartner et al. 2022), thus it can be argued that increased deposition of brown fat could be positively related to survival in cold conditions. The mechanisms involved are difficult to study in vivo (Hergenhan 2012), although infrared thermography has been identified as a technology suitable for the measurement and discrimination of levels of thermogenesis in lambs (McCoard et al. 2014; Labeur et al. 2017; Vicente-Pérez et al. 2019; Cai et al. 2023).

Studies suggest prenatal shearing and cold exposure during late pregnancy can enhance lamb thermoregulation by increasing brown fat reserves and non-shivering thermogenesis, improving cold resistance (Stott and Slee 1985; Symonds et al. 1992; Symonds and Lomax 1992). However, other studies have not been able to replicate improved thermoregulatory abilities in lambs after prenatal shearing of the ewes (Kenyon et al. 2002a, b).

This study aimed to examine whether acute husbandry-like stressors and/or cold stress, typical of shearing, at different stages of pregnancy in pregnant Merino ewes, affect neonatal lamb thermoregulation, using infrared thermography. We hypothesised that lambs born to prenatally stressed ewes would display improved thermoregulatory ability during a one-hour cold challenge.

## Materials and methods

Three experiments compared the effects on the neonate thermoregulation of: mid-pregnancy shearing to handling as sham-shearing; the effects of ewe cold exposure during late-pregnancy; and the impact of a combination of acute stressors and cold exposure to ewes during mid or late-pregnancy. Lamb thermoregulation was assessed on four-hour-old lambs using infrared thermography images (ThermaCam T640, FLIR Systems AB, Danderyd, Sweden) taken at 10-minute intervals during a 1-hour cold challenge at 4˚C in an environment controlled cold room (± 15 min).

Weather conditions (ambient temperature and relative humidity) were obtained from CustomWeather (Time and Date AS 1995–2017. All rights reserved) for each experiment during the treatment and lambing periods. Temperature ranges for each period were calculated using the lowest daily minimum and the highest daily maximum recorded during that period, while relative humidity was averaged over each period.

### Experimental animals

Experiments were conducted at CSIRO Chiswick, Armidale, Australia (30.52°S, 151.67°E, 1050 m). The site is located in the Northern Tablelands of New South Wales and experiences a cool‑temperate climate, characterised by cold winters, mild to warm summers, and moderate seasonal rainfall, with the warmest conditions typically occurring in late spring and summer. Ewes were sourced from the research farm, health-checked daily, and identified using livestock-marking spray (Steadfast Stockmark, Dy-Mark, Australia).

*Experiment 1* used 30 pregnant multiparous Merino ewes and 34 lambs. Ewes were included in the experiment and selected on pregnancy status at 80 days after ultrasound scanning. The 30 ewes were divided into two treatment groups: mid-pregnancy (day 90 of pregnancy) shearing followed by wetting (SH) and mid-pregnancy handling as a sham treatment (HA). Ewes had been shorn during gestation the previous year and carried about one year’s wool growth when prenatal treatments were applied. Gestation lengths ranged between 139 and 156 days, as calculated from estimated day of pregnancy on the date of scanning. There were 15 ewes per group which resulted in the assessment of 17 SH lambs (9 singles and 8 twins) and 18 HA lambs (7 singles, 8 twins and 3 triplets).

*Experiment 2* initially had 61 pregnant multiparous Merino ewes that had been naturally mated. They were in their last two weeks of pregnancy (pregnancy day 130) when divided into two treatment groups; a wet and cold exposed (CE) and a control group (TR). Ewes had been shorn during gestation the previous year and carried 13-month wool growth when prenatal treatments were applied. Gestation lengths ranged between 142 and 164 days. Ultimately, due to experimental challenges, including a power outage that disrupted video recording of lambing events and lambing occurring outside the planned period (likely due to inaccuracies in foetal aging at scanning), the number of lambs available for the cold challenge was limited. Of those born during the trial, only18 CE (16 singles and 2 twins) and 19 TR lambs (13 singles and 6 twins) from 17 CE and 16 TR ewes were assessed in the cold challenge component.

*Experiment 3* used 108 pregnant mature Merino ewes selected on their pregnancy status at 80 or 110 days based on ultrasound scanning. The ewes were subdivided into three treatment groups: a mid-pregnancy (pregnancy day 88–92) stressed group (MID), a late-pregnancy (pregnancy day 117–123) stressed group (LATE) and a control group (CTRL). Ewes had been shorn prior to joining and carried about 3–4 month wool growth when prenatal treatments were applied. Gestation lengths ranged between 130 and 146 days. Seventy-eight lambs were tested from these three groups, 27 CTRL lambs (13 singles and 14 twins), 25 LATE lambs (9 singles and 16 twins) and 24 MID lambs (16 singles and 8 twins).

### Housing and feed

When in the paddocks, in *Experiments 1 and 2*, ewes grazed improved native pasture (Feed-on-offer > 1000kgDM/ha) and were supplemented with 150 g/day/ewe sheep pellets (metabolisable energy 10.5 MJME/kgDM, 17.5% crude protein, 2.5% fat). In *Experiment 3* ewes grazed pasture improved native pasture (feed-on-offer > 1000kgDM/ha) and supplemented with 100 g/animal/day of maize grain (~ 13MJME/kgDM, ~ 9% crude protein) to meet maintenance requirements. While feed‑on‑offer was not directly quantified, pasture availability was sufficient throughout the experimental periods and was not considered limiting to intake. Feed intake was managed according to standard sheep feeding recommendations for pregnant Merino ewes, based on CSIRO nutrient requirement guidelines, with supplement intake adjusted to ensure pregnancy requirements were met and that animals were not nutritionally restricted. Pasture availability was sufficient throughout the experimental periods and was not considered limiting to intake.

Across all experiments, nutritional management aimed to maintain ewe Body Condition Score between 2.8 and 3.3 (0 being emaciated, 5 obese), in line with best practice guidelines (Gibb and Treacher 1982; Everett-Hincks and Dodds 2008; Kenyon et al. 2012), with condition assessed using subsampling at handling events and ongoing visual monitoring.

When indoors, in *Experiment 1*, during the 7-day treatment period and the preceding 5-day acclimation period, ewes were housed indoors in group pens (3 m² per ewe) on slatted floors, separated by treatment group. During the treatment period, ewes were fed 400 g of pellets and 900 g of 50:50 lucerne and oaten chaff mix per ewe per day.

For all three experiments, approximately 10 days before lambing, ewes were moved indoors into an open-sided multipurpose shed with lambing pens, onto straw bedded pens with 3–4 ewes per pen (1.5 m^2^ per ewe) and kept under continuous artificial lighting 24 h per day. Ewes were visually checked at least every 30 min and monitored through a video surveillance system (Hikvision) using IVMS4200 software connected to 10 Hikvision EXIR Bullet True Day and Night vision network cameras (Hikvision Digital Technology Co, Hangzhou 310052, China). During lambing, ewes received a 50:50 lucerne and oaten chaff mix (0.65 kg/ewe/day) supplemented with sheep pellets (as described above; 0.65 kg/ewe/day for single‑bearing ewes; 0.85 kg/ewe/day for twin‑bearing ewes) and maize grain (200 g/ewe/day).

### Treatments

#### Experiment 1 - Shearing and handling stressors

On the first day of the experiment (day 0), the treated group was shorn (SH) while the control group was sham-shorn for two minutes (HA), by mimicking the handling associated with shearing (seated on haunches, manual stroking on both sides of the body and rump). In the 7 days post shearing, the SH ewes, but not HA ewes, were wetted using ceiling sprinklers for 30 min (water temperature 8–10˚C, ambient temperature 5–12 ˚C), on three days (days 0, 3 and 7). Treatment took place 7–14 August 2014 with temperatures ranging from 0 °C to 15 °C and relative humidity at 63%.

#### Experiment 2 - Cold exposure stressor

During the last two weeks of pregnancy (from day 135 of pregnancy), ewes assigned to the CE group were transported and cold exposed (CE) on days 0, 2, 5 whereas the treatment group (TR) were transported only. Each event included a 10 min loading and transport (5 km) from the paddock to the cold room facility using a stock trailer. At the cold room facility, CE ewes were unloaded and individually wetted for two minutes using a high-pressure hose with cold water sourced from the main water supply. During the wetting, ewes were seated on their rump to mimic shearing, ensuring wool-free areas around the legs and groin were thoroughly soaked. The CE ewes were then held in a 4˚C cold room for 3 h, without forced air movement. Concurrently, the TR ewes were unloaded and held in an undercover slatted floor pen (ambient 15–21 °C). At the completion of the cold treatment all animals were transported back to their paddock. Treatments were conducted from 26 November to 23 December 2014 under mild conditions (10–29 °C; 75% humidity).

#### Experiment 3 – Combination of stressors

From day 90 (MID) or day 125 (LATE) of pregnancy both treatment groups (MID and LATE) were exposed to a stress treatment on five occasions (days 0, 2, 5, 8 & 11). The stress treatment mimicked all stress components of shearing, including cold stress: 10 min of yarding, 10 min of transport in a stock trailer, and 10 min of high-water pressure hosing, followed by three hours in a 4 °C cold room. During this treatment, the control group (CTRL) remained in a paddock (ambient temperature 6–12 °C). Treatment took place from 15 July to 5 August 2015 (–5 °C to 14 °C; 85% humidity) for mid-pregnancy and 19 August to 9 September (–4 °C to 18 °C; 76% humidity) for late-pregnancy.

### Lambing

All lambs born alive during the experiments were ear-tagged within an hour of birth and those without precise birth times were excluded from the study. Cold challenge and thermoregulation assessment were conducted 4 h after birth to allow ewe-lamb bonding. The cold challenge and thermoregulation assessment period, including transport to and from the cold challenge, lasted approximately 70 min.

In *Experiment 1*, of 30 ewes, 25 lambed during the experiment and a total of 35 lambs were included with two cases of dystocia observed. In *Experiment 2*, of 61 ewes, 36 lambed during the experiment and a total of 38 lambs were included with six cases of dystocia observed. In *Experiment 3*, of 108 ewes, 60 lambed during the experiment, a total of 78 lambs were included, and one case of dystocia was observed. Dystocia was defined as foetopelvic disproportion or malpresentation preventing the lamb to exit the pelvis and identified when no progress in lambing was observed after two hours. When observed, lambing was assisted, and lambs were visually monitored after birth to confirm successful suckling of colostrum. Five lambs (2 in *Experiment 1–* HA x1 and SH x1, 1 in *Experiment 2* – CE x1, 2 in *Experiment 3* – CTRL x1, LATE x1) with poor suckling were deemed too weak to undergo cold challenge and excluded from the study. They died within 24 h despite colostrum, heat, and resuscitation attempts. *Experiment 1*, lambing occurred from 29 September to 17 October 2014 (2–24 °C; 56% humidity), between 6 December and 21 December 2014 and continuing into 5–13 January 2015 (8–28 °C; 77% humidity) for *Experiment 2*. Finally lambing for *Experiment 3* for both groups occurred between 14 and 24 September 2015 (0–19 °C; 74% humidity).

#### Cold challenge

**Fig. 1:**
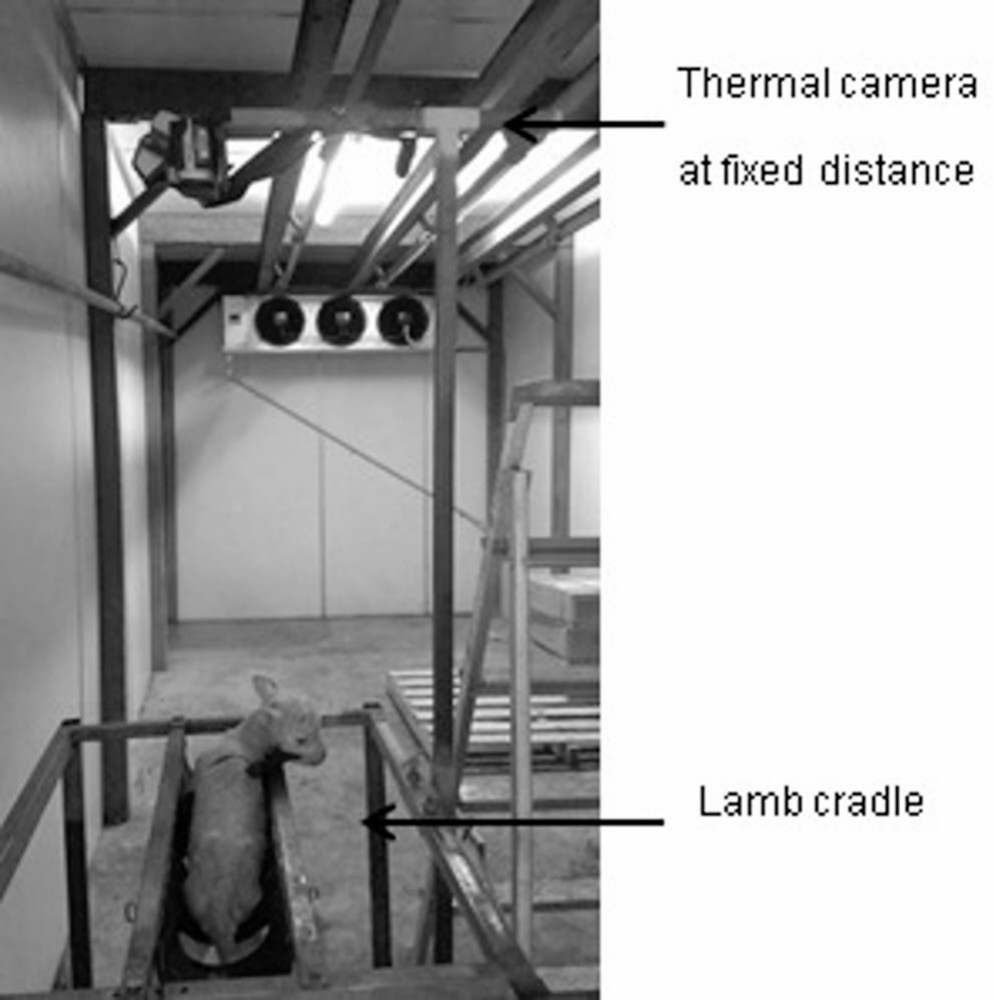
Cold room set up showing cradle used to restrain lambs during a cold challenge of up to 60 min and the mount for the infrared thermography camera 1 m above the back of the lamb as per Labeur et al. (2017). Fans were turned off when animals were present

Four hours after birth, lambs were transported in a 68-litre plastic carrier box (Ezy Storage, Australia), minimising human contact, to a temperature-controlled room. The wool length of each lamb, measured at the midline of the dorsal region, midway between the shoulders and the hips, was classified (‘short’ < 3 mm/’medium’ 3–6 mm [only included in *Experiment 1* and subsequently removed]/’long’ > 6 mm) and their abdominal, inguinal and axillar areas were wetted (tap water in half-filled 20 L bucket; temperature 8–10 ˚C) to mimic amniotic fluid cover typically present immediately after birth. After being wetted, lambs were then placed inside a 4 ˚C cold room (without fan-forced air movement) and held in a cradle to limit movement (Fig. 1). If required, lambs were secured with a bandage around the shoulder girdle. Following cold challenge, lambs were returned to their mothers, and ewe-lamb behaviour monitored for up to 60 min to ensure the ewe-lamb bond was re-established.

### Thermography and monitoring of body temperature

During the cold challenge, thermograms were taken every 10 min (T0-T60) using an infrared thermography camera (ThermaCam T640, FLIR Systems AB, Danderyd, Sweden; methodology by Labeur et al. (2017). To monitor for hypothermia, rectal temperatures were measured every 10 min with a digital thermometer (accuracy ± 0.1 °C from 35 to 42 °C; Flexible Rapid 10 s Thermometer DT_K101A; Medshop Australia, Preston VIC) at the same interval. If rectal temperature dropped below 36.5 °C, lambs were removed from the cold room and given warm milk. In total, 18 lambs were removed from the cold challenge prior to completing 60 min under this protocol (for *Experiment 1*, 7 HA, 9 SH; for *Experiment 3*, 1 CTRL and 1 LATE; none for *Experiment 2*). For these lambs, data until the point of removal was included in the analysis.

### Thermogram analysis

**Fig. 2:**
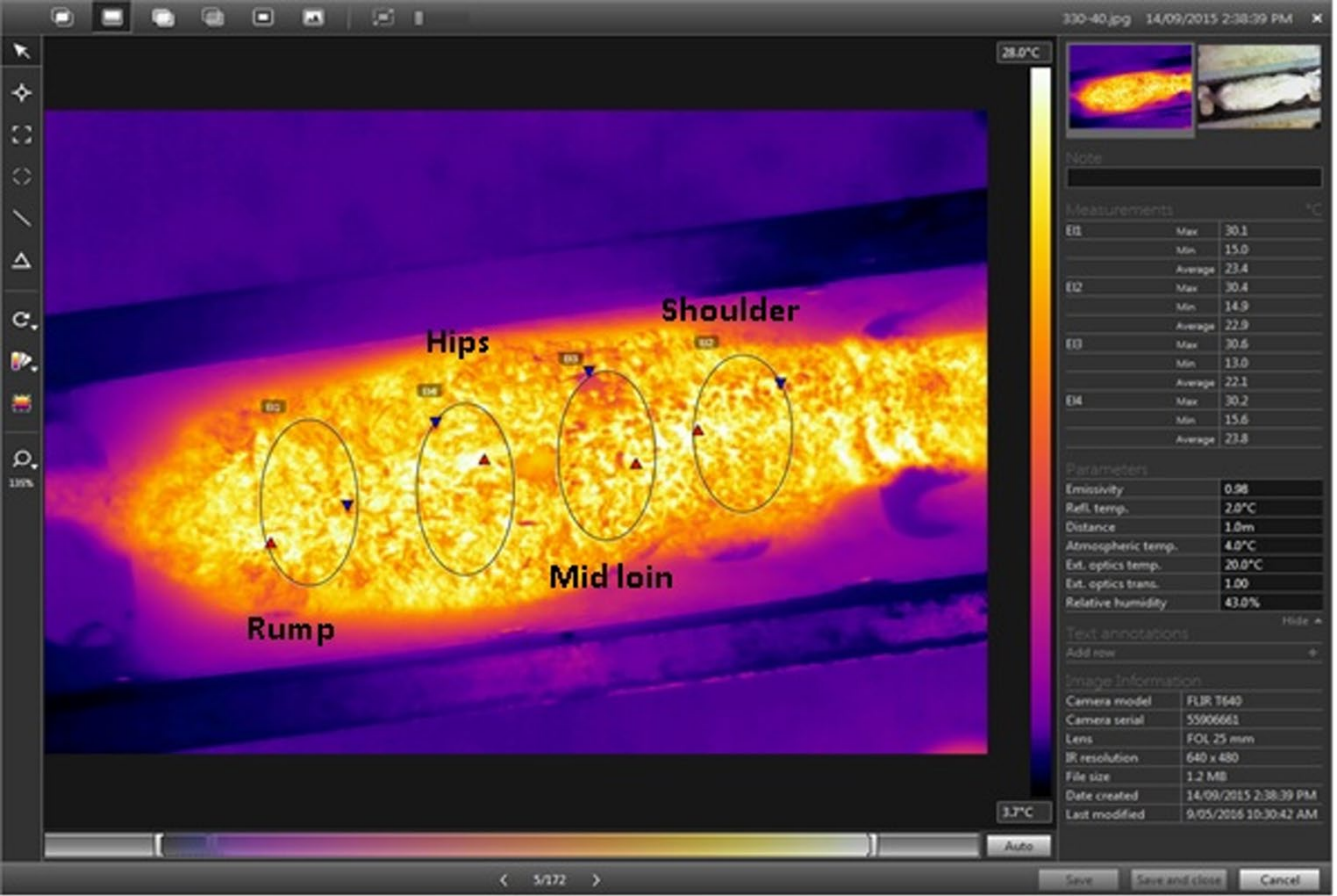
Screenshot of thermogram from FLIR Tool software, displaying the arrangement of the 4 zones across the back of the lamb, shoulder, mid loin, hips and rump of a lamb as described by Labeur et al. (2017). In the right panel, the radiated body surface temperature values for each zone are displayed, minimum, maximum and average of which only the last two were recorded

Thermograms were analysed in FLIR Tools (FLIR Systems AB), as per Labeur et al. (2017). For each picture, average and maximum radiated temperatures were extracted from four fixed zones (shoulder, mid loin, hips and rump; Fig. 2). If the lamb was secured with a bandage around the shoulder girdle, data from the shoulder zone could not be exported.

### Statistical analysis

Average and maximum radiated body surface temperature (RadTemp) were analysed using repeated measures ANOVA mixed models (nlme package (Pinheiro et al. 2013) in R (v3.6.0, The R foundation for Statistical Computing). Treatment, zone, timepoint, wool length, litter size, sex and their interactions were included as fixed effects and lamb ID as a random effect. Zone-specific comparisons used similar mixed models.

For each lamb, the variable ∆T_60_=T60-T0 represented the change in RadTemp from baseline (T0) to 60 min (T60). Additionally, as for some lambs the cold challenge was terminated at 40 min (T40) due to hypothermia (6 HA lambs, 7 SH lambs and 2 MID), ∆T_40_=T40-T0 was calculated using the same method as ∆T_60_. ΔT variables were analysed using mixed models with the same fixed and random effects as described above. Normality of distribution was assessed via a Shapiro-Wilk test with a 5% threshold and Box Cox transformations were applied where needed. Models initially included body weight or girth circumference as covariates to account for lamb size but these and other non-significant factors (sex, zone-time and treatment-sex interactions) were subsequently removed. Dystocic lambs were included in the analysis as none of these lambs showed evidence of hypothermia during the cold challenge.

Results are expressed as least squares means (LSmeans) ± standard deviation (S.D.). Statistical significance was set at *P* ≤ 0.05; 0.05 < *P* < 0.1 indicated a trend toward significance.

## Results

### Experiment 1 - Shearing and handling stressors

**Fig. 3:**
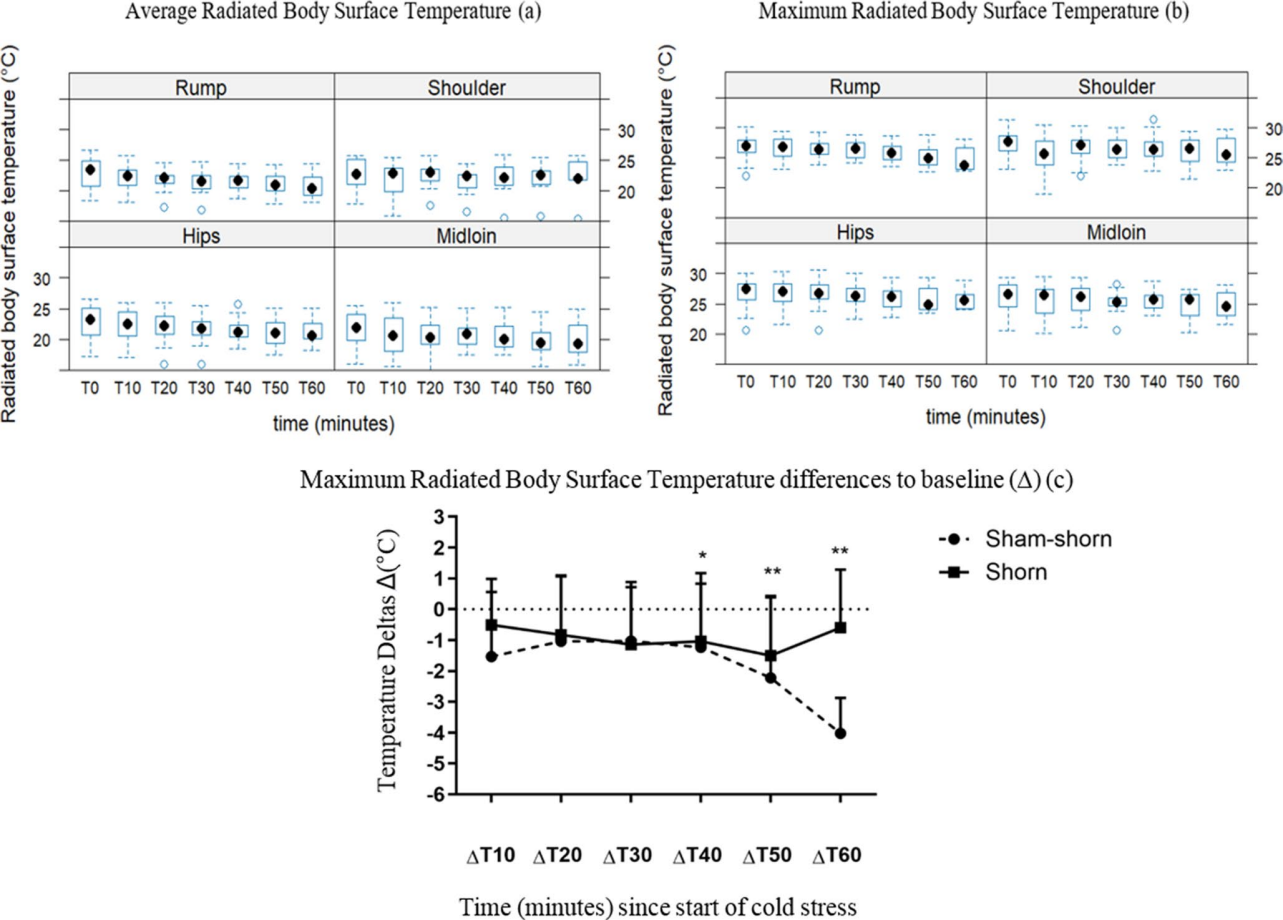
Average (**a**) and maximum (**b**) radiated body surface temperature profiles for four zones on lambs’ backs (shoulder, mid-loin, hips, rump) recorded every 10 min during a 1-hour cold challenge for lambs born to ewes shorn (SH) or sham-shorn (control; HA) in mid-pregnancy. (**c**) Change in maximum lamb body surface temperature (temperature deltas ∆) from baseline at each time point during the cold challenge, for lambs born to ewes in each treatment group (SH and HA). *0.05 ≤ *P* < 0.1; **P < 0.05

Average and maximum RadTemp decreased over time similarly pattern across all body zones (Fig. 3 (a) & (b)). Lambs born to SH ewes tended to have higher maximum RadTemp than HA lambs at ‘Mid loin’ (*P =* 0.073; 26.24 vs. 25.14 °C). A similar effect occurred for ∆T_60_Max (*P =* 0.025) and a tendency for ∆T_40_Max (*P =* 0.072; Fig. 3 (c)).

Across timepoints and treatments, average RadTemp was significantly higher at ‘Hips’ than ‘Shoulder’ (*P =* 0.013) and ‘Shoulder’ tended to be lower than ‘Rump’ (*P =* 0.08). However, ‘Shoulder’ was significantly higher than ‘Mid loin’ (*P <* 0.001) and ‘Rump’ was higher than ‘Mid loin’ (*P <* 0.001).

Maximum RadTemp was lower at ‘Mid loin’ than ‘Hips’, ‘Rump’ and ‘Shoulder’ (*P <* 0.001) and ‘Rump’ tended to be lower than ‘Shoulder’ (*P =* 0.035).

Wool length significantly impacted RadTemp: lambs with short wool (< 3 mm) had significantly higher temperatures (average: 23.08 ± 0.50 °C, maximum: 26.86 ± 0.33 °C) than long wool (≥ 6 mm) lambs (average: 18.45 ± 1.20 °C, maximum: 25.05 ± 0.85 °C) (*P* < 0.05).

### Experiment 2 - Cold exposure stressor

**Fig. 4:**
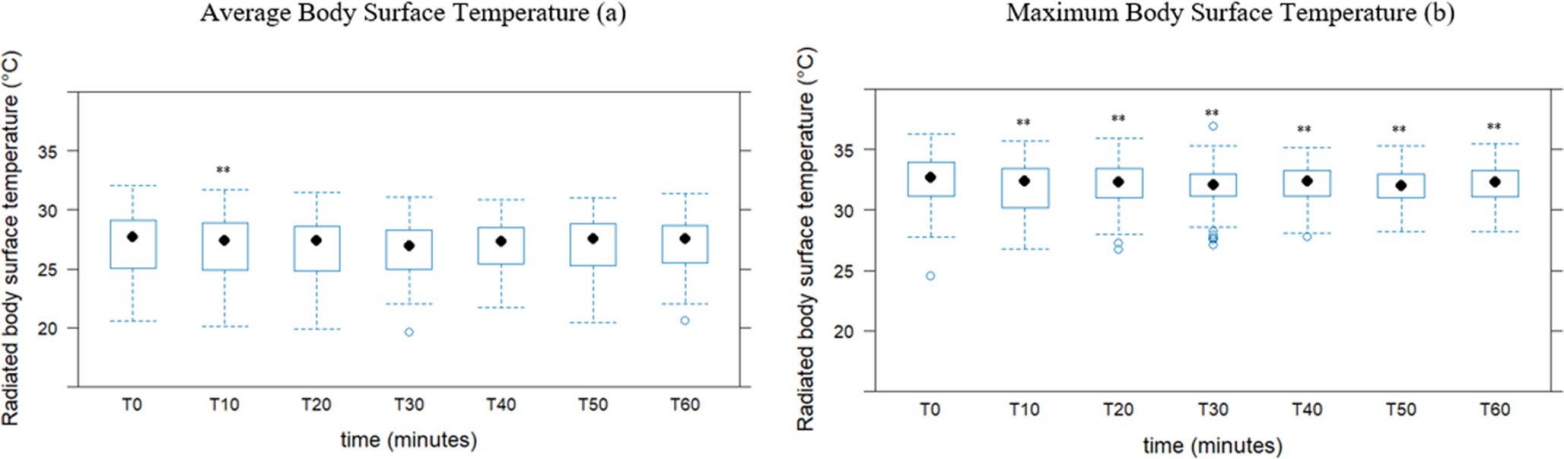
(**a**) Average and (**b**) Maximum radiated body surface temperature profiles during a 1-hour cold challenge for lambs born to both ewes transported wetted and cold exposed (CE) during late-pregnancy and ewes transported only (control; TR) during late-pregnancy. ** *P* < 0.05 for timepoints when compared with body surface temperature at start

Across all four zones, average RadTemp was higher at T0 than T10 (*P =* 0.0439; Fig. 4(a)) and maximum RadTemp at T0 exceeded all other time points (*P <* 0.01; Fig. 4(b)). In CE lambs, ‘Rump’ average RadTemp was higher than ‘Mid loin’ and ‘Shoulder’ (respectively, 27.58 ± 0.73 vs. 26.50 ± 0.73 °C; *P <* 0.001 and 26.99 ± 0.76 °C; *P =* 0.049), but this was not observed in TR lambs. No other treatment or wool length effects were detected.

### Experiment 3 – Combination of stressors

**Fig. 5:**
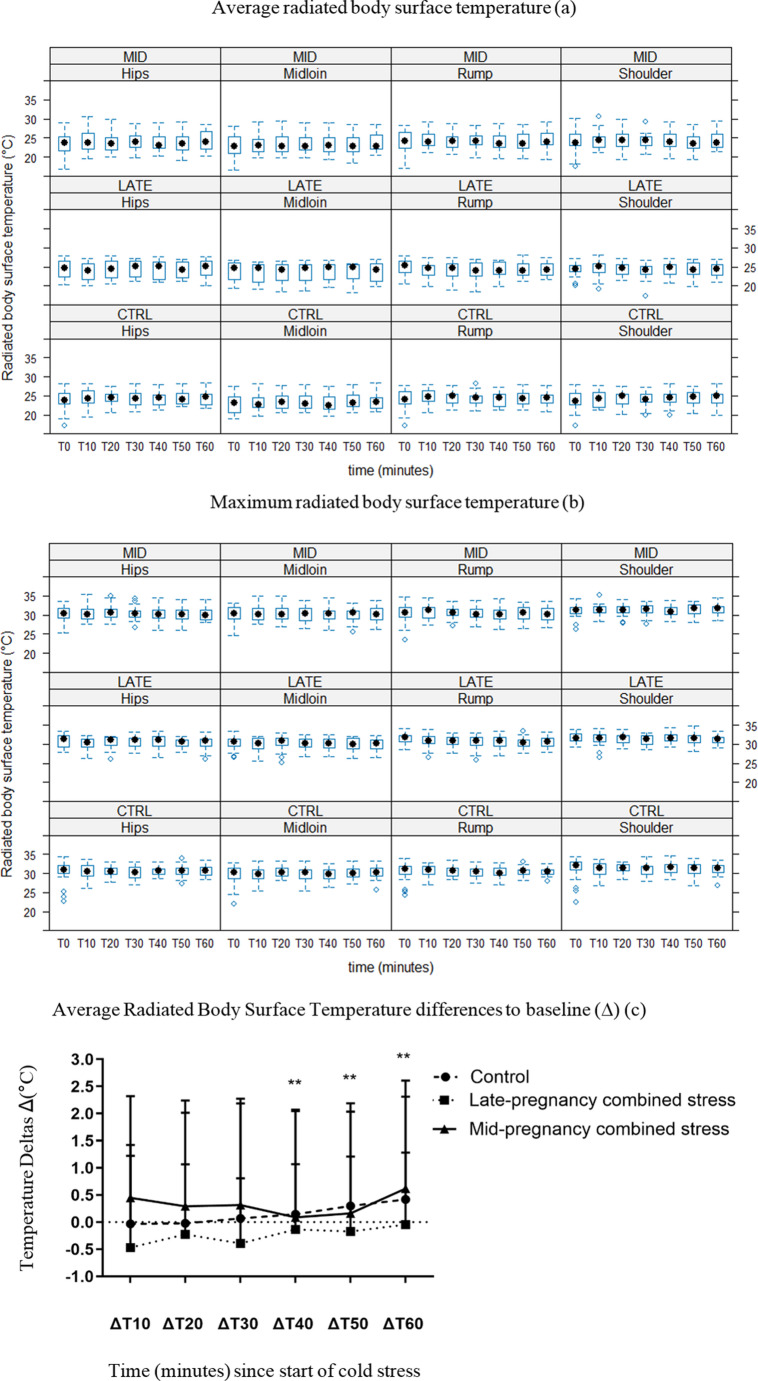
Average (**a**) and maximum (**b**) radiated body surface temperature profiles for four zones on the back of lambs (Shoulder, Mid loin, Hips, and Rump) during a 1-hour cold challenge for lambs born to ewes that were yarded, transported, and cold-exposed during mid-pregnancy (MID), late-pregnancy (LATE), or were untreated controls (CTRL). (**c**) Change in average lamb body surface temperature (temperature deltas ∆) from baseline at each time point during the cold challenge, for lambs born to ewes in each treatment group (MID, LATE and CTRL).**P < 0.05

Across timepoints and combined treatments, average RadTemp was higher at ‘Hips’ than ‘Mid loin’ (*P <* 0.01) but lower than ‘Shoulder’ (*P =* 0.042; Fig. 5(a)). ‘Rump’ average RadTemp was higher than ‘Mid loin’ (*P <* 0.01) and tended to be lower than at ‘Shoulder’ (*P =* 0.06). Maximum RadTemp showed a similar zone pattern (*P <* 0.001; Fig. 5(b)).

At ’Rump’, LATE lambs had higher maximum RadTemp than MID (*P <* 0.05) and higher average RadTemp than CTRL (*P <* 0.05). Average RadTemp for ∆T_40_ was greater for MID lambs than LATE (*P =* 0.01) or CTRL (*P =* 0.0017; Fig. 5(c)) with the same pattern for ∆T_60_ (Fig. 5(c)).

No time effect was observed for maximum RadTemp but average RadTemp at T0 was higher than other timepoints for MID and CTRL (*P <* 0.05).

Short wool lambs had higher maximum RadTemp (*P <* 0.0001) and average RadTemp than long wool lambs at all zones (Table 1). Single lambs had higher maximum RadTemp than twins at ‘Rump’, ‘Mid loin’ and ‘Hips’ (*P <* 0.001).

**Table 1:** Average and maximum radiated body surface temperatures across all timepoints (from 0 to 60 min) and treatments (ewes exposed to combined husbandry and cold stress during mid-pregnancy, late-pregnancy, and control) for four zones (‘Rump’, ‘Shoulder, ‘Hips’, Mid loin’) during a 1-hour cold challenge for lambs with short (< 3 mm) and long wool (> 6 mm). LS-Means for maximum radiated body temperatures are back-transformed

Zone	Value	Short wool (< 3 mm)	Long wool (> 6 mm)
Rump	Average (°C)	24.93 ± 0.25 ^b^	22.92 ± 0.35 ^a^
	Maximum (°C)	31.05 ^b^	29.88 ^a^
Shoulder	Average (°C)	25.05 ± 0.23 ^b^	23.12 ± 0.32 ^a^
	Maximum (°C)	31.73 ^b^	30.8 ^a^
Hips	Average (°C)	25.07 ± 0.26 ^b^	22.65 ± 0.37 ^a^
	Maximum (°C)	30.96 ^b^	29.7 ^a^
Mid loin	Average (°C)	24.33 ± 0.28 ^b^	21.69 ± 0.39 ^a^
	Maximum (°C)	30.7 ^b^	29.17 ^a^

Different superscripts within a row indicate significant differences (*P*<0.0001)

## Discussion

This study examined how acute husbandry stressors, such as transport, yarding and shearing and cold stress, individually or combined, at different stages of pregnancy, affect newborn thermoregulation during a 1-hour cold challenge. Our findings suggest that both shearing and combined stressors produced similar effects on lamb body surface temperature patterns, suggesting improved thermoregulation.

Prenatal husbandry-like stress combined with cold exposure produced lambs with increased body surface temperature and better maintenance of their radiated body surface temperature during the cold challenges than control lambs (*Experiments 1* & *3)*. *Experiment 2* showed no difference in radiated body surface temperature, likely due to small sample size. It may also suggest that cold exposure was not the sole contributor to improved body surface temperature maintenance in treatment lambs. Alternatively, because ewe body temperature was not monitored during cold exposure, ewes in *Experiment 2* may not have been truly cold stressed, and the treatment may not have elicited a physiological response sufficient to influence lamb thermoregulation. Additionally, as ewes were in full wool, the insulating effect of wool may have further mitigated the intended impact of cold stress (Slee and Ryder 1967). Stott and Slee (1985) reported a greater thermogenic response to cold challenge in lambs born to ewes cold stressed during late-pregnancy (14 days pre-lambing), supporting this interpretation. In our study, improved body surface temperature maintenance resulted from combined cold exposure and husbandry-like stressors, consistent with observations by Symonds et al. (1992) and Clarke et al. (1997), where chronic late-pregnancy cold stress, from winter shearing and fleece removal, enhanced lamb brown fat thermogenesis. Differences in prenatal stress intensity and duration may also explain outcome variation. Our stressors were transient and short term, contrasting with previous studies using prolonged cold exposure to mimic winter conditions (Clarke et al. 1997; Stott and Slee 1985; Symonds et al. 1992). Nonetheless, the consistency between shearing alone and combined stress treatment in improving lamb thermoregulation suggests activation of similar physiological pathways.

Timing of prenatal stress appeared to have little influence on lamb thermoregulation.

In this study, mid-pregnancy shearing was associated with higher and more stable lamb body surface temperatures, reflected by a smaller decrease in surface temperature (ΔT), compared with lambs born to ewes handled similarly but without wool removal during a cold challenge. Similarly, acute stress and cold exposure applied during both mid- and late-pregnancy resulted in higher body surface temperatures compared with controls, which may reflect increased endogenous heat production, potentially associated with greater brown adipose tissue activity.

While most prior studies focused late-pregnancy and the last month of pregnancy, little is known about the potential effects of stress during mid-pregnancy. This emphasis reflects that brown fat matures rapidly in the final weeks of gestation, its rate of accumulation slows and its thermogenic capacity peaks at birth (Alexander 1978; Gemmell and Alexander 1978). Cold exposure during pregnancy alters maternal and foetal metabolic profiles, raising maternal glucose and non-esterified fatty acids and increasing foetal glucose concentrations (Thompson et al. 1982), which could influence brown fat development. However, shearing also triggers hormonal and metabolic changes in ewes (Clarke et al. 1997), including increased cortisol (Hargreaves and Hutson 1990; Mears et al. 1999), and elevated plasma non-esterified fatty acid (Elvidge and Coop 1974). Both mid- and late-pregnancy shearing have been associated with prolonged elevations of the thyroid hormones T3 and T4 (Symonds et al. 1988; Sherlock et al. 1999), which are critical for brown fat activation and thermoregulation (Bienboire-Frosini et al. 2023). Finally, maternal cold exposure induces peripheral vasoconstriction to conserve heat, which may reduce adipose tissue blood flow and limit fat deposition in the ewe, potentially altering nutrient availability for the foetus (Thompson et al. 1982). Therefore, acute stress and cold exposure at different stages of pregnancy likely act through different mechanisms. While late-pregnancy stress may affect maternal glucose levels and vasoconstriction, affecting brown fat maturation and deposition, mid-pregnancy stress may elevate foetal T3 and T4 levels.

From this study, conclusions cannot be drawn on the mechanism underlying differences in lamb thermal response to prenatal treatments. Infrared thermography only measures heat loss through the skin and body surface, which reflects heat production and thermoregulation. Our aim was to assess heat production across different areas of the body and determine whether areas near brown fat depots might show greater activity. Based on patterns reported by Labeur et al. (2017), we used four fixed-size zones (shoulder, mid loin, hips and rump) along the lamb. Across all experiments, temperature trends were consistent, with the lowest reading at ‘Mid-loin’. Although brown fat depots are concentrated in peri-renal, abdominal and inguinal regions (Symonds 2013), the greater sensitivity observed at the mid-loin position is likely explained by significant peri-renal depots (Labeur et al. 2017).

Wool length also influenced results in both shearing and stress-combination experiments. which aligns with previous report that skin coverage such as wool impacts infrared thermography reading (Mota-Rojas et al. 2021). Wool provides thermal insulation, reducing heat loss and supporting metabolic mechanisms for core temperature maintenance (McCutcheon et al. 1983; Martin 1999; Allain et al. 2010). In our study, short-wool lambs showed greater heat loss than long-wool lambs, likely due to long-wool lambs having fewer thermal windows, which are poorly insulated regions where heat loss occurs, making direct skin temperature readings difficult (McCafferty et al. 2011).

In all experiments, the body surface temperature of each lamb dropped within the first 10 min of the cold challenge starting, initiating non-shivering thermogenesis (Gunn et al. 1991).

## Conclusion

In conclusion, prenatal stress influenced the ability of newborn lambs to maintain body surface temperature during cold exposure. Prenatal cold exposure combined with husbandry-related stressors impacted heat production in newborn lambs and altered body surface temperature responses, regardless of whether stress occurred in mid- or late-pregnancy. Observable effects at both stages suggests at least two alternate mechanisms may be involved, one regulating brown fat activity via thyroid hormones, and another increasing total brown fat deposition in the lamb.

Future research should include longer cold challenges to confirm the onset of non-shivering thermogenesis and continuous core body temperature monitoring to clarify links between body surface temperature and thermogenic response. Studies should also examine heat loss, hormonal profiles and brown fat deposition in parallel to better understand their interplay and refine interpretation of infrared thermography data.

## Data Availability

The data of this study are available from the corresponding author upon reasonable request.
